# Blind Spoofing GNSS Constellation Detection Using a Multi-Antenna Snapshot Receiver †

**DOI:** 10.3390/s19245439

**Published:** 2019-12-10

**Authors:** Johannes Rossouw van der Merwe, Alexander Rügamer, Wolfgang Felber

**Affiliations:** Satellite Based Positioning Systems Department, Fraunhofer IIS, Nordostpark 84, 90411 Nuremberg, Germany; alexander.ruegamer@iis.fraunhofer.de (A.R.); wolfgang.felber@iis.fraunhofer.de (W.F.)

**Keywords:** GNSS, beamforming, server-based processing, antenna array

## Abstract

Spoofing of global navigation satellite system (GNSS) signals threatens positioning systems. A counter-method is to detect the presence of spoofed signals, followed by a warning to the user. In this paper, a multi-antenna snapshot receiver is presented to detect the presence of a spoofing attack. The spatial similarities of the array steering vectors are analyzed, and different metrics are used to establish possible detector functions. These include subset methods, Eigen-decomposition, and clustering algorithms. The results generated within controlled spoofing conditions show that a spoofed constellation of GNSS satellites can be successfully detected. The derived system-level detectors increase performance in comparison to pair-wise methods. A controlled test setup achieved perfect detection; however, in real-world cases, the performance would not be as ideal. Some detection metrics and features for blind spoofing detecting, with an array of antennas, are identified, which opens the field for future advanced multi-detector developments.

## 1. Introduction

Spoofing, the transmission of false signals to manipulate a receiver, is a significant threat to the security, reliability, and integrity of the global navigation satellite system (GNSS) users [1,2]. A spoofer aims to manipulate the position, velocity, and time (PVT) solution of the receiver and is consequently associated with criminal, terrorism, and military activities. Transmitting, and by extension spoofing, in unallocated radio frequency (RF) spectrum, or transmitting without permission is illegal, according to international spectral policies and agreements [3,4]. However, this does not deter spoofing attacks [5]. There are many anti-spoofing methods and schemes to detect spoofing [2,6,7,8]; however, many of these fail when a spoofing attack is more sophisticated (e.g., a synchronized attack) or cooperative (i.e., the owner of the receiver assists with the attack) [9]. A transmitter in a spoofing attack is usually terrestrial [5]. Therefore, the spatial distribution over the open-sky of real GNSS signals is not emulated. Consequently, spatial detection methods have proven a continued success, independent of the spoofing method used [10,11]. Spatial detection methods require either the utilization of an array of antennas or a highly directional antenna [12]. Most commonly, an array of antennas that is time and frequency-synchronized is required, such that detection methods, like direction-of-arrival (DOA) estimation, can be applied.

This article applies array-based spoofing detection methods to server-based GNSS processing methods. Server-based GNSS processing allows for the remote evaluation of GNSS signals, based on a signal recording (i.e., baseband samples) made by a receiver [13]. As the processing is done remotely, it is also referred to as cloud GNSS [14], or snapshot processing, as a small snapshot of baseband or intermediate frequency data is sent from the receiver to the server for processing [15,16]. In turn, the server computes the PVT solution of the snapshot. One aim of this technique is to remove the processing burden from the receiver and to transfer it to the server. Server-based processing can be applied to low-power devices [17,18], which cannot afford a full GNSS receiver for positioning. This saves on power and weight requirements for the receiver as well as the associated system. Hence, it is often used for low size, weight and power (SWAP) devices. Applications such as remote sensing and animal tracking can benefit from this technique [19]. Mobile devices can also profit from this approach [13]. Another application for server-based processing is the verification of a receiver’s position using encrypted GNSS signals [20,21]. Inexpensive receivers typically do not have the required security module to allow cryptographically protected GNSS signals to be used on-board, but a server containing this security module can outsource the capability without exposing sensitive information. A snapshot of data may be sent to this server for authentication. Snapshot receivers have a limited window of data. Therefore, many popular temporal spoofing detection methods are not applicable [9]. This further emphasizes the need for spatial detection methods for snapshot-based receivers.

This article presents multiple spoofing detection methods using an antenna array in conjunction with a snapshot receiver. It extends on the previous snapshot-based blind spoofing detection methods [22], by considering a system of detectors to improve the overall detector performance. An exploratory approach is used with empirical analysis, to identify suitable detection metrics. The snapshot concept aims to transfer the processing load to the server and to maintain receiver complexity and requirements as low as possible. Therefore, the benefits of array processing can be applied to SWAP devices. The drawback of using multiple antennas is that it requires synchronized receiver channels. This increases the system complexity and cost which makes this technique counter-productive for low SWAP devices. However, it can be argued that this is a necessity for reliable spoofing detection. DOA based detection requires the array to be calibrated, which further increases the cost and complexity of the system. The focus of this research was to develop and assess blind detection methods which do not require DOA, thereby reducing the receiver complexity, cost, and the need for array calibration.

A background on snapshot receivers, server-based processing, and the implementation of beamforming to improve performance is provided in Section 2. The spoofing detection algorithm for a single pair of spoofed satellites is presented in Section 3, with additional theoretical analysis to support performance expectations. An expansion to system-level detection methods is done in Section 4. The experimental setup is introduced in Section 5, results are presented in Section 6, and discussed in Section 7. Finally, conclusions are drawn in Section 8.

## 2. Snapshot Receivers and Beamforming

A snapshot receiver operates with a few milliseconds of raw data obtained by the analog-to-digital converter (ADC) output following the RF front-end [15]. This raw data snapshot is sent to a server for processing. As there are limited data available, the server can only achieve signal acquisition: there are not enough data available for a tracking process, nor sufficient data to obtain any information from the navigation message. Since the ephemeris cannot be decoded, this information is obtained from a secondary source. A direct pseudorange cannot be derived from the acquisition results since the transmission time of the satellite is not available. However, given a rough receiver position and time estimate together with the ephemeris data, pseudorange reconstruction can be achieved [23]. Once the PVT has been calculated and verified, the result might be sent back to the receiver, depending on the architecture and needs of the receiver.

### 2.1. Acquisition Signal Model

Acquisition forms the base of the entire processing chain—not just for initialization like with a conventional receiver. Therefore, it is important to have high performance during this stage. Since the transport channel between the receiver and the server is often limited, the snapshot size is reduced to satisfy data transmission requirements. This is a trade-off for the receiver, since the smaller the snapshot is, due to length, sample-rate, and quantization reduction, the poorer the acquisition performance [24]. Acquisition performance with snapshot receivers using multiple antennas has been investigated in a previous study [25]. This method has shown good performance, as it uses both spatially incoherent and spatially coherent integration (i.e., beamforming) to improve acquisition performance.

The received signal system x(t) can be described as:

(1) x(t)=A×s(t)+n(t),

where x(t) is the received signal for each antenna element, represented as a number of antenna elements $N_{e}$ sized column vector; n(t) is a $N_{e}$ sized noise vector and is assumed to be additive white Gaussian noise (AWGN); A is a $N_{e}\times N_{s}$ matrix containing the array vectors for each of the $N_{s}$ signals. This A matrix is defined as:

(2) $$A=\left[a_{1},a_{2},\cdots ,a_{N_{s}}\right]\;,$$

where $a_{i}$ is a column vector of size $N_{e}$ describing the complex array coefficients. These are a function of the DOA of the signal, the position of the antenna elements, the gain of the receiver channels, and the phase offset for the receiver channels. The received signal vector s(t) is a $N_{e}\times 1$ vector, where each element represents a different GNSS signal form a different satellite. For the *i*-th satellite, the received signal is defined as:

(3) $$s_{i}\left(t\right)=\alpha_{i}\cdot r_{i}\left(t-\tau \right)\cdot d_{i}\left(t-\tau \right)\cdot e^{2\pi j\cdot f_{D}t}\;,$$

where $\alpha_{i}$ is the complex gain for the signal (i.e., amplitude and phase); τ is the code phase (i.e., the time offset); $r_{i}\left(t\right)$ is the pseudo random noise (PRN) code for the specific satellite; $d_{i}\left(t\right)$ is the modulated data on the signal and is—dependent on the signal type—a combination of the navigation message and the secondary code [26]; and $f_{D}$ is the Doppler frequency offset induced by the relative movement between the satellite and the receiver. To isolate a single signal, the acquisition stage correlates the received signal with a replica. The replica typically contains a single iteration of the PRN code. In general, PRN codes are selected to have good cross- and auto-correlation properties; hence, the correlation enhances the correct signal while suppressing other signals, interference, and noise. As an example, a single received signal will be:

(4) $$y_{i,j}\left(t\right)=s_{i}\left(t\right)⋆r_{j}\left(t\right)=\left\{\begin{array}{cc} \alpha_{i}\cdot d_{i}\left(t\right)\cdot R_{i,i}\left(\tau \right)\tau_{\mathrm{int}}\sin c\left(\tau_{\mathrm{int}}f_{D}\right) & \mathrm{if}\;i=j\\ 0 & \mathrm{if}\;i\neq j \end{array}\right.\;,$$

where “⋆” is the correlation function; $R_{i,i}\left(\cdot \right)$ is the cross ambiguity function (CAF) for the given PRN sequence; $\tau_{\mathrm{int}}$ is the integration time used during correlation; and sinc(·) is the sinc-function, also called the sampling function [27]. From this equation, it is clearly illustrated how the PRN sequence can isolate the selected signal. Further, if the code phase offset τ and the Doppler offset $f_{D}$ is zero, the correlation will have a maximum value. To find these values, which are the maximum correlation peak, is the purpose of the acquisition processing. This is done by modulating the replica with different Doppler frequencies and by shifting the replica code with different time delays.

Typically, the longer the integration time $\tau_{\mathrm{int}}$ is, the more processing gain is achieved, which results in better performance and noise suppression. However, this is limited by the bit-transitions of the modulated data $d_{i}\left(t\right)$. In the worst case, a bit-transition can result in deconstructive self-interference, which will completely remove the signal during the correlation stage. As such, either the bit-transitions need to be estimated or incoherent integration is required. As the PRN sequence r(t) is periodic in time, the correlation output $y_{i,j}\left(t\right)$ is also quasi-periodic: the magnitude of the correlation function is periodic in time, but the phase is not, due to the modulated bit-stream. To combine these values incoherently in time, the squared values are summed:

(5) $$y_{i,j}^{N_{\mathrm{inco}}}\left(t\right)=\sum_{l=0}^{N_{\mathrm{inco}}-1}y_{i,j}\left(t-l\cdot T_{\mathrm{rep}}\right)\times y_{i,j}^{*}\left(t-l\cdot T_{\mathrm{rep}}\right)=\sum_{l=0}^{N_{\mathrm{inco}}-1}{∥y_{i,j}\left(t-l\cdot T_{\mathrm{rep}}\right)∥}^{2}\;,$$

where $N_{\mathrm{inco}}$ is the number of temporal incoherent epochs to use, $T_{\mathrm{rep}}$ is the repetition interval for the PRN code, ${\left(\cdot \right)}^{*}$ is the complex conjugate of the function, and $∥\cdot ∥$ is the magnitude function. As these values are added incoherently, longer effective integration times can be achieved. However, due to the incoherent addition, there is also a squaring loss. This makes this method less effective in comparison to temporal coherent integration, but it is significantly more robust to bit-transitions.

### 2.2. Spatial Acquisition Techniques

Combining all spatial channels coherently results in improved acquisition performance [25]. This is also referred to as beamforming or array processing [28]. It can be done before or after correlation [29,30], referred to as pre- and post-correlation beamforming. Typically, pre-correlation beamforming is preferred, as this is more processing efficient than post-correlation beamforming since fewer correlation operations are required. An array steering vector is used to combine the data from each of the channels. The function of the array steering vector is to correct the amplitude and phase offsets from each channel, such that signals are aligned and can be coherently combined. An array steering vector $b_{i}$ is a column vector of size $N_{e}\times 1$. Pre-correlation beamforming can be described as:

(6) $$y_{i,j}^{\mathrm{pre}}\left(\tau ,f_{D}\right)=\left(b_{i}^{T}\times x\left(t\right)\right)⋆r_{j}\left(t-\tau ,f_{D}\right)\;,$$

and post-correlation beamforming as:

(7) $$y_{i,j}^{\mathrm{post}}\left(\tau ,f_{D}\right)=b_{i}^{T}\times \left(x\left(t\right)⋆r_{j}\left(t-\tau ,f_{D}\right)\right)\;,$$

As both these correlation processes and the matrix multiplications are linear, these operations are per definition associative. Therefore, the outputs of both functions are mathematically equal. The disadvantage of coherent spatial integration is that the array steering vector $b_{i}$ should be known. This can either be estimated or be determined theoretically if sufficient a-priori information is available. An alternative approach is incoherent spatial integration. This follows a similar approach to incoherent temporal integration, but instead of exploiting the periodicity of the signal, it exploits the temporal similarities between the antenna channels. Once again, the correlation functions have peaks at the same locations; however, with different phases. Incoherent spatial integration for $y_{j}^{\mathrm{inco}}\left(\tau ,f_{D}\right)$ can be described as:

(8) $$y_{j}^{\mathrm{inco}}\left(\tau ,f_{D}\right)=\sum_{i=1}^{N_{e}}∥x_{i}\left(t\right)⋆r_{j}\left(t-\tau ,f_{D}\right)∥\;.$$

Using spatial incoherent acquisition does not suppress interferences or other signals. Further, it also has a squaring loss. Hence, it is less effective against noise in comparison to coherent beamforming. However, it allows the correlation peak to be found more reliable than only using a single channel. Once the corrected code phase τ and Doppler offset $f_{D}$ is found, the array steering vector can be determined. Let the correlation output vector $y_{i}\left(\tau ,f_{D}\right)$ for each channel be defined as:

(9) $$y_{i}\left(\tau ,f_{D}\right)=x\left(t\right)⋆r_{i}\left(t-\tau ,f_{D}\right)\;.$$

If the code phase τ and Doppler offset $f_{D}$ are correctly estimated, it can be shown to be equal to:

(10) $$y_{i}\left(\tau ,f_{D}\right)=a_{i}\circ \alpha_{i}\cdot R_{i,i}\left(0\right)+n_{c}\;,$$

where ∘ is the Hadamard product, and $n_{c}$ is the noise vector after correlation. From these values the array steering vector can be simply estimated:

(11) $$b_{i}={\left(y_{i}\left(\tau ,f_{D}\right)\right)}^{\circ -1}\;,$$

where ${\left(\cdot \right)}^{\circ -1}$ is the Hadamard inverse (i.e., an element wise inverse). Note that in this case the array steering vector also contains the corrections to the receiver phase and amplitude errors ($\alpha_{i}$). Hence, these do not need to be estimated separately. More sophisticated methods to determine the array steering vector can also be implemented [31,32,33,34]. Lastly, if incoherent temporal integration was used, like with Equation (5), then the average phase and magnitude offsets relative to the first element is done. This removes the bit-transitions in the code as well:

(12) $$b_{i}=\sum_{l=0}^{N_{\mathrm{inco}}-1}{\left(y_{i}\left(t-l\cdot T_{\mathrm{rep}},f_{D}\right)\right)}^{\circ -1}\cdot e^{2\pi j\cdot ∠y_{1,i}\left(t-l\cdot T_{\mathrm{rep}},f_{D}\right)}\;,$$

where “∠” is the angle operator.

### 2.3. Combined Acquisition Strategy

**Figure 1:**
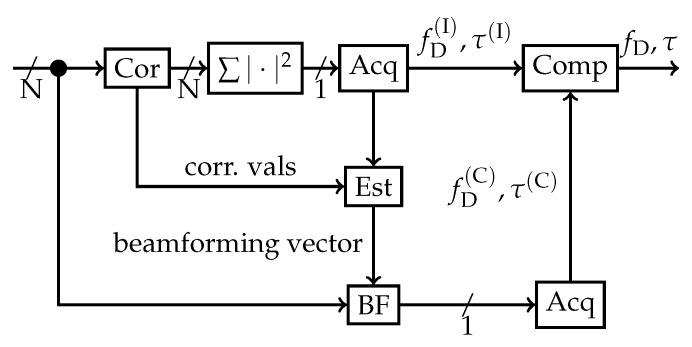
Block diagram for a blind beamforming acquisition method. ©IEEE. Reprinted, with permission, from [22].

An effective blind acquisition technique, which firstly implements incoherent spatial integration to find the correct correlation peak, secondly estimates the array steering vector, and thirdly does coherent spatial integration, has already been developed [25]. This method is completely blind, as no DOA estimation is done. Figure 1 shows the method that was used for acquisition and to estimate the array steering vector. Consequently, no calibration or information about the array configuration or orientation is required. The acquisition method first applies incoherent spatial acquisition followed by coherent spatial acquisition with an estimated array steering vector to improve performance. As this application focuses primarily on beamforming, the array steering vector is also referred to as the beamforming steering vector or the beamforming weights. Hence, these terms in this scenario can be used interchangeably.

Firstly, blind incoherent acquisition is carried out for each receiver channel. The received signals from every array element are correlated simultaneously with the replica of the current satellite (denoted by “Cor”). The absolute squared values of the correlations are added (“${\sum |\cdot |}^{2}$”) in order to estimate the code phase $\tau^{\left(I\right)}$ and carrier Doppler $f_{D}^{\left(I\right)}$ in the acquisition stage (“Acq”). If the satellite is acquired, then the array steering vector is estimated (“Est”) with the code phase $\tau^{\left(I\right)}$ and carrier Doppler $f_{D}^{\left(I\right)}$ from the incoherent acquisition results. Once the vector is estimated, the receiver channels are weighted accordingly (denoted by “BF”) and a second acquisition takes place. This time the coherent $\tau^{\left(C\right)}$ and carrier Doppler $f_{D}^{\left(C\right)}$ are determined. After the second acquisition stage, the code phases and Doppler frequencies from the first (incoherent), and the second (coherent) acquisitions are compared. If there is a significant difference, the satellite will be discarded and not used after acquisition. This allows a secondary test to remove false positives from the acquisition process. This double acquisition is processing-intensive, but it has shown high reliability and false acquisition suppression in previous studies [25].

A limitation of this method is that the estimation of the array steering vector is based upon the results of incoherent acquisition. These values may contain multi-path or cross-correlation components from other signals, which can obscure the estimation process. In turn, the erroneous array steering vector may form a beam that does not sufficiently suppress these unwanted signal components. This raises concerns, especially in harsh environments with significant multi-path components, as it may result in offsets in the acquisition estimates, which in turn result in a degraded PVT solution.

## 3. Single Detection

Spoofing detection with an array of antennas has been proven successfully [11,35,36]. However, this requires a calibrated array, known array orientation, known receiver phase offsets, and a synchronized receiver. These methods are DOA based and estimate the direction to each satellite. As the snapshot concept aims to outsource the processing to the server, it implicitly shifts the cost of the system away from the receiver. However, all calibrations required for array processing would increase teh cost and complexity of the receivers. One solution to counteract high complexity and cost is to use blind array processing. Further, most array steering methods are also applied to conventional receivers where successive and recursive estimation on a continuous signal is available.

A drawback of blind methods is that the detection is relative: a single signal originating from the wrong direction cannot be detected. However, if multiple signals originate from the same direction, they can be detected. Since a constellation of signals is usually spoofed, this would not be an issue in real-life scenarios. However, in the case of distributed spoofing attacks (i.e., multiple spoofing transmitters) [37], it would be significantly more difficult to detect. Fortunately, distributed attacks are rare, due to the associated difficulty [7,37].

The spoofing detection algorithm presented in this article uses the array steering vector obtained in acquisition (see Section 2), as this information is already available and can be used. The array steering vector is tested for similarity by correlating each pair of vectors with each other. If the signals originate from the same direction, then they have similar vectors and have a high correlation value. The detector is based on this correlation value and implements a detection for each pair of array steering vectors.

The detector implements a detection for each pair of steering vectors. Hence, a system of detections is developed. This makes the detection process more efficient. First, all the estimated array steering vectors $b_{n}$ are stacked in a matrix B:

(13) $$B=\left[b_{1},b_{2},\ldots ,b_{N_{s}}\right]\;,$$

where $b_{n}$ is a column vector for the array steering vector for the *n*-th satellite. The constellation consists of a total of $N_{s}$ satellites. B is a number of elements $N_{e}$ by the number of estimated steering vectors $N_{s}$ sized matrix. The matrix is correlated to get a non-normalized correlation matrix C:

(14) $$C=B^{H}\times B\;,$$

where ${\left(\cdot \right)}^{H}$ is the Hermitian transpose of the signal and “×” is a matrix multiplication. Each element in the matrix is equivalent to the dot product between each pair of steering vectors. C is a $N_{s}$ by $N_{s}$ Hermitian matrix. To normalize the matrix, the magnitude values of the auto-correlations need to be determined:

(15) $$c=\sqrt{\mathrm{diag}\left(C\right)}\;,$$

where c is a column vector containing the magnitude values, and $\mathrm{diag}\left(\cdot \right)$ takes the diagonal of a matrix. The normalized correlation matrix $C_{\mathrm{norm}}$ is calculated as:

(16) $$C_{\mathrm{norm}}=ℜ\left\{C\right\}\circ {\left(c\times c^{T}\right)}^{\circ -1}\;,$$

where $ℜ\left\{\cdot \right\}$ takes the real component of the correlation, ∘ is the Hadamard product, and ${\left(\cdot \right)}^{\circ -1}$ is the Hadamard inverse.$C_{\mathrm{norm}}$ is also an Hermitian matrix. Each element of this matrix has the form:

(17) $$C_{\mathrm{norm}}\left(n,m\right)=\frac{ℜ\left\{b_{n}^{*}\cdot b_{m}\right\}}{∥b_{n}∥∥b_{m}∥}=\cos\left(\xi_{n,m}\right)\;,$$

where $∥b_{n}∥$ is the magnitude of the *n*-th beamforming vector. Each correlation can be regarded as the cosine of the angle $\xi_{n,m}$ between two array steering vectors. This is not the spatial angle of the DOA between two satellites (also referred to as the incident angle of the array), but a measure of how similar the two steering vectors are. For the remainder of this article, this angle will be referred to as the steering vector angle.

As an example, a uniform linear array (ULA) with an inter-element spacing of half a wavelength has beamforming steering vectors in the form of:

(18) $$b={\left[e^{\;j\pi \cdot 1\cdot \sin\theta },e^{\;j\pi \cdot 2\cdot \sin\theta },\ldots ,e^{\;j\pi \cdot N_{e}\cdot \sin\theta }\right]}^{T}\;,$$

where θ is the spatial angle (incident angle) to the broadside of the array. For a two-element array, the steering vector can be proven to have the form:

(19) $$\cos\left(\xi_{1,2}\right)=\cos\left(\xi_{2,1}\right)=\cos^{2}\left(\frac{\pi }{2}\sin\theta \right)\;.$$

In this case, the steering vector angle $\xi_{2,1}$ diverges rapidly from the spatial angle θ (array incident angle).

**Figure 2:**
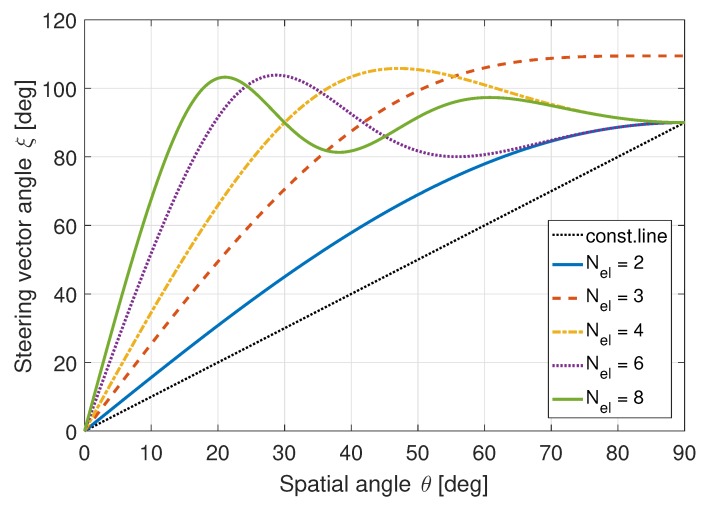
Spatial angle θ vs. steering vector angle $\xi_{m,n}$ for a ULA. ©IEEE. Reprinted, with permission, from [22].

Figure 2 shows the expected behavior for ULAs with different number of elements. As the number of elements increases, the beamforming angle separates quicker with smaller spatial angles. Consequently, with more elements used, increased diversity can be exploited for detection.

**Figure 3:**
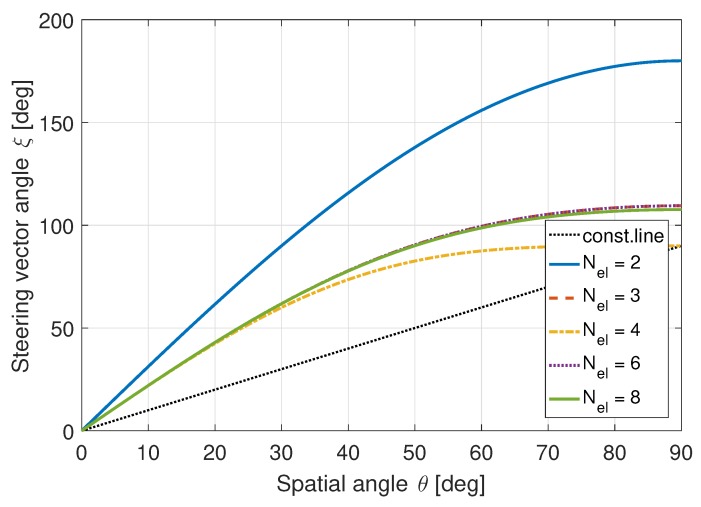
Spatial angle θ vs. steering vector angle $\xi_{m,n}$ for a UCA. ©IEEE. Reprinted, with permission, from [22].

ULAs are straightforward to analyze. However, they are not suitable for GNSS applications as these antenna arrays only have beamforming capabilities in a single dimension. GNSS signals can originate from any direction above the horizon; hence, two-dimensional beamforming capabilities are required. Therefore, uniform circular arrays (UCAs) are often used for GNSS receivers [38]. As the beamforming properties of arrays are dependent on the array configuration, the performance of this method will be fundamentally different. Figure 3 shows the expected behavior for a UCA under similar circumstances.

The previous two figures only show the ideal case of one signal being on the broadside of the antenna array and the other signal moving relative to it. ULAs and UCAs have ambiguities: two satellites at different locations can have similar steering vectors. The result is that these signals would seem to be originating from the same direction and would be detected and classified as spoofed signals. In order to reduce the false detection probability due to array ambiguities, the cumulative distribution function (CDF) for all the difference angles are calculated. The CDF is used to summarize the statistics for different array orientations, as the array steering vector is dependent on the array geometry, orientation, and incident angle. Hence, the CDF represents the overage likelihood that the difference value is below a certain value. More details of CDFs and statistical distributions are presented in Section 5.

**Figure 4:**
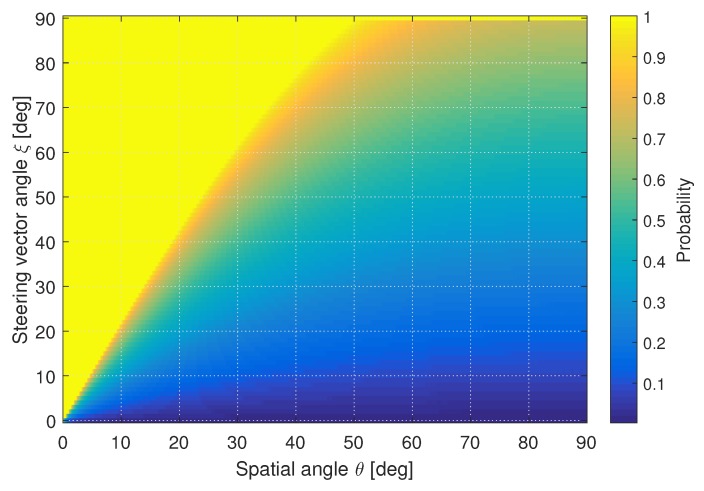
CDF of spatial angle θ vs. the steering vector angle $\xi_{m,n}$ for a UCA. ©IEEE. Reprinted, with permission, from [22].

Figure 4 shows the CDF function of the steering vector angle for a UCA consisting of six elements. The test angles are for a single cross-section consisting of all elevation angles and a single azimuth value. The angles to which the CDF first achieves a value equal to one (i.e., 100% certainty), follows the ideal case as shown in Figure 3. However, it can also be seen that there are many cases where the steering vector angle is spread which may result in poor performance in these cases.

This analysis shows that the separation between the spatial and steering vector angle can be used as a good detector to determine how similar the origins of two signals are. The detector threshold is tuned according to a symbolic steering vector separation angle. This is the steering vector angular threshold $\xi_{\mathrm{Th}}$:

(20) $$\lambda_{\mathrm{Th}}=\cos\left(\xi_{\mathrm{Th}}\right)\;.$$

This symbolic angle provides an intuitive understanding of how the detector functions, as opposed to merely setting an arbitrary threshold value. The threshold is used to detect each value in the matrix $C_{\mathrm{norm}}$. If the value is lower than the threshold then the null-hypothesis $H_{0}$ of no spoofing is accepted. Otherwise, the alternative hypothesis $H_{1}$, where the signals have similar origins and are considered spoofed, is accepted:

(21) $$C_{\mathrm{norm}}\left(n,m\right)=\cos\left(\xi_{n,m}\right)\underset{H_{1}}{\overset{H_{0}}{≷}}\lambda_{\mathrm{Th}}\;.$$

A detection matrix D can be generated accordingly:

(22) $$D\left(n,m\right)=\left\{\begin{array}{cc} 1, & \mathrm{if}\;C_{\mathrm{norm}}\left(n,m\right)\leq \lambda_{\mathrm{Th}}\\ 0, & \mathrm{if}\;C_{\mathrm{norm}}\left(n,m\right)>\lambda_{\mathrm{Th}} \end{array}\right.\;.$$

Both normalized the correlation matrix $C_{\mathrm{norm}}$ and the detection matrix D are used in the subsequent sections to develop system-level detectors. As such, obtaining these matrices are the first step for the subsequent blind detection methods.

## 4. System of Detections

In the previous section, single detections between satellite similarities were done. In this section, three strategies to combine the matrix of results to improve spoofing detection are presented. These strategies result in different metrics and features which can later be used for detection or further statistical approaches.

### 4.1. Fraction of Detections

The first method is to consider the number of detections in the upper triangle of the detection matrix D. The size of the detection matrix D depends on the number of successfully acquired satellites $N_{s}$. The total number of detection values *N* in the upper triangle is:

(23) $$N=\frac{N_{s}\left(N_{s}-1\right)}{2}\;.$$

The detector observes the number of detections in this triangle. If at least *M* detections are made, then the system is selected as being spoofed. If it is assumed that all of the satellites are either completely spoofed or only real signals (i.e., no combined real and spoofed signals), the detection probabilities for the system can be determined to be:

(24) PD(M)=∑m=MNNmPd|SmPd¯|SN−m,

where Nm is the binomial coefficient, $P\left(d|S\right)$ is the conditional probability that a value is detected *d*, given that the signals are spoofed *S*. The inverse is indicated by $\bar{d}$ (i.e., it is not detected). Similarly, the probability of a missed detection $P_{M}^{\left(M\right)}$, the probability of false alarm $P_{FA}^{\left(M\right)}$, and the probability of a correct rejection $P_{R}^{\left(M\right)}$ can also be determined:

(25) PM(M)=∑m=0M−1NmPd|SmPd¯|SN−m

(26) PFA(M)=∑m=MNNmPd|S¯mPd¯|S¯N−m

(27) PR(M)=∑m=0M−1NmPd|S¯mPd¯|S¯N−m

This approach improves overall performance as it uses each combination of the satellites to verify whether the complete constellation is spoofed or not. Thereby, false detections from spatially similar satellites can be efficiently suppressed. Further, by combining all of the detections the overall system improvement allows for increased tuning freedom within individual detection.

One issue of this approach of defining a minimum number of detection, is that the total number of detectors *N* is inconsistent. The total number of detectors depends on the size of the matrix $C_{\mathrm{norm}}$, which in turn is dependent on the number of satellites that were successfully acquired. To overcome this, a fraction of detections $\frac{M}{N}$ is used. This is called an *M* of *N* detector [39], but also referred to as binary integration [40,41] or coincidence detection [42,43]. A fraction of detections $\frac{M}{N}$ approach makes the system independent on the number of detectors to evaluate, as it is normalized. For example, if at least half of the values should be detected, then the system detector threshold is defined as $\frac{M}{N}=\frac{1}{2}$. If too few detections are made then the null-hypothesis $H_{0}$ of no spoofing is accepted. Otherwise, the alternative hypothesis $H_{1}$ is accepted, where the signals have similar origins and are considered spoofed. This can be formally defined as:

(28) $$\lambda_{\mathrm{frac}}=\frac{M}{N}\underset{H_{1}}{\overset{H_{0}}{≷}}\frac{\sum_{n,m}D\left(n,m\right)-N_{s}}{2N}\;,$$

where D(i,j) is the *i*-th row and the *j*-th column of the detection matrix D (described in Section 3), and $\lambda_{\mathrm{frac}}$ is the detector threshold for the fraction of detections metric. It can further be simplified using Equation (23):

(29) $$\lambda_{\mathrm{frac}}\underset{H_{1}}{\overset{H_{0}}{≷}}\frac{\sum_{n,m}D\left(n,m\right)-N_{s}}{N_{s}\left(N_{s}-1\right)}\;.$$

The fraction of detection is a simple yet effective method of combining multiple detectors.

### 4.2. Eigenvalue Based Detection

Eigenvalue methods can be used for detection [44,45]. Eigenvalue decomposition separates a matrix to a set of orthogonal Eigenvectors and Eigenvalues. The magnitude of each Eigenvalue describes the contribution of the associated Eigenvector to the original matrix. The Eigenvalues are the characteristic roots of the system [46], whereby the distribution of the Eigenvalues describes the coherency of the elements of the linear system they represent. It directly links to the rank of the matrix. As such, these can be effectively used to detect the monotonicity of the matrix. Eigenvalue decomposition is described as:

(30) $$A=Q\Lambda Q^{-1}\;,$$

where A is the matrix to be decomposed, Q is a matrix where each column is a unique Eigenvector, and Λ is a diagonal matrix containing the Eigenvalues. For the remainder of the analysis, it is assumed that the Eigenvalues are placed in descending order. The maximum Eigenvalue to the mean of the Eigenvalues is used as a detection method [47]:

(31) $$\lambda_{\mathrm{eig}}\underset{H_{1}}{\overset{H_{0}}{≷}}\frac{∥\lambda_{1}∥}{\frac{1}{N}\sum_{i}∥\lambda_{i}∥}=\frac{∥\Lambda (1,1)∥}{\frac{1}{N}∥\Lambda ∥}\;,$$

where $\lambda_{\mathrm{eig}}$ is the detection threshold, $\lambda_{1}$ is the largest Eigenvalue, $\lambda_{i}$ is the *i*-th Eigenvalue from the matrix, and *N* is the number of Eigenvalues. If the largest Eigenvalue is similar in size to the mean of the Eigenvalues and consequently below the threshold, the null-hypothesis $H_{0}$ of no spoofing is accepted. Otherwise, the alternative hypothesis $H_{1}$ is accepted.

### 4.3. Clustering and Community Based Detection

As spatial spoofing detection evaluates the similarity of the array steering vectors, a simple expansion thereupon is to attempt to cluster the array steering vectors together. This is a measure of how well the satellites can be grouped. The Louvian clustering algorithm is an iterative algorithm that optimizes the modularity of a data set [48,49]. The data set consists of several nodes and edges (links) between these nodes. Highly connected nodes are grouped into communities by this algorithm. This means that correlated nodes would be grouped in communities making this algorithm ideal for identifying “spoofing communities”. This allows the algorithm to identify which subset of satellites are spoofed and which are the real GNSS signals. At the same time, the modularity of the data set is an indication of the connectedness of the nodes. Hence, high modularity indicates that the nodes can be grouped efficiently into communities, whereas low modularity indicates that the data is highly random and not simply grouped into communities. Further, as the nodes are grouped into communities, the possibility to detect multiple spoofing transmitters is also possible (i.e., where a spoofer uses two or more antennas to transmit a subset of the signals). However, this phenomenon requires further investigation and is suggested for future research.

This algorithm requires a matrix A which contains the weight of the edges between each of the nodes. The higher values in this matrix corresponds to stronger links between the nodes. This is represented by the column and row of the matrix. Further, this algorithm requires a vector c which contains a list of the different communities.

Maximizing the modularity of the data set achieves effective clustering. The modularity *Q* is defined as:

(32) $$Q=\frac{1}{2m}\sum_{i,j}\left(A_{ij}-\frac{k_{i}k_{j}}{2m}\right)\delta \left(c_{i},c_{j}\right)\;,$$

where *m* is the total connectedness between all of the nodes:

(33) $$m=\frac{1}{2}\sum_{i,j}A_{ij}\;,$$

and $k_{i}$ is the connectedness of the *i*-th node to the other nodes and is defined as:

(34) $$k_{i}=\sum_{j}A_{ij}\;.$$

Lastly, δ(·) is a modified Dirac function and is defined as:

(35) $$\delta \left(c_{i},c_{j}\right)=\left\{\begin{array}{cc} 1, & \mathrm{if}\;c_{i}=c_{j}\\ 0, & \mathrm{otherwise} \end{array}\right.\;.$$

At the start of the clustering process, all of the nodes are placed in separate communities. The initial modularity *Q* for the system is calculated. Systematically, each node is then moved to different communities. The community which results in the greatest increase of system modularity *Q* for each node is then selected as the new community for that node. When all of the nodes have been adjusted to the optimal community clustering, a new system is formed by grouping nodes from the same community. Thereby, implicitly reducing the dimensions of the matrix A in each iteration. Thereafter, the process is repeated until no improvement of the modularity *Q* is achievable. As the algorithm iterates, similar communities are clustered together to form new and larger communities.

To apply the Louvain algorithm to the spoofing values, the normalized correlation matrix $C_{\mathrm{norm}}$ or the detection matrix D can be selected as the edge matrix A. Using the normalized correlation matrix $C_{\mathrm{norm}}$ will have more dynamics and information about the values; however, it would require more processing. Using detection matrix D will be faster, but as small matrices are used (the Louvain algorithm was developed to address millions of nodes), this improvement in processing requirements is negligibly small. The modularity *Q* of the system, the number of communities that are formed, and the difference in community sizes within a system, are possible metrics that can be exploited for detector development.

The most promising one seems to be the modularity *Q*. If the system can be effectively clustered, i.e., all the satellites are closely related and assumed to be spoofed, it would have a low modularity *Q*. As such the detection problem can be formulated as:

(36) $$\lambda_{\mathrm{mod}}\underset{H_{0}}{\overset{H_{1}}{≷}}Q\;,$$

where $\lambda_{\mathrm{mod}}$ is the detection threshold for the modularity. If the values are above the threshold, the system cannot be clustered effectively. Hence, the system is diverse and the null-hypothesis $H_{0}$ of no spoofing is accepted. Otherwise, the alternative hypothesis $H_{1}$ is accepted, where the system is spoofed. Clustering forms an intuitive approach to spatial spoofing detection, hence, it forms part of the new detection metrics proposed by this article.

## 5. Experimental Setup and Methodology

**Figure 5:**
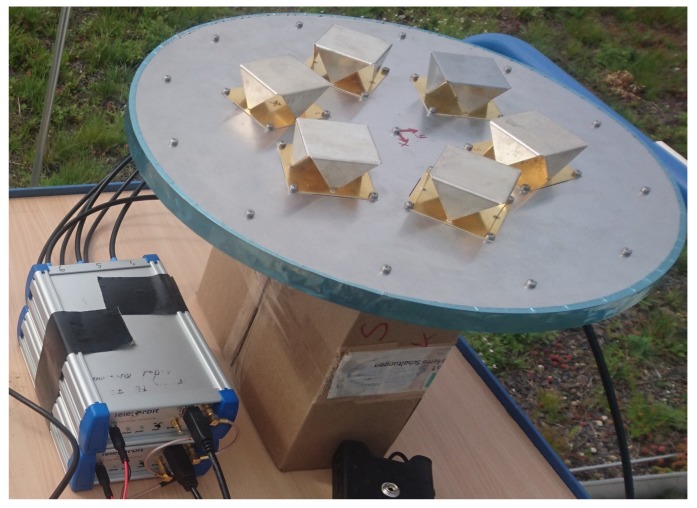
Photo of the measurement system. ©IEEE. Reprinted, with permission, from [22].

Two experimental setups were used: the first one used a six-element UCA. The array was connected to a six-channel receiver which consisted of two synchronized three-channel Flexiband front-ends [50], synchronized with a 10 MHz clock and triggered simultaneously. These front-ends have a maximum sample-rate of 81 MHz at 8 bit I/Q; however, for this experiment, a common sampling rate of 10.125 MHz was used. One minute of data has been recorded and then evaluated in post-processing. A picture of the system is shown in Figure 5. The recording was made under open-sky conditions with the purpose to determine the probability of false alarm for the developed detectors.

The second setup used a single antenna connected to each channel of the receiver via a splitter. For this recording, all signals will have similar and static array steering vectors. Therefore, this was considered a rudimentary spoofing simulation, without the need for an anechoic chamber or a live setup. With this recording, the probability of detection for the detectors was evaluated.

Only Global Positioning System (GPS) L1 C/A signals were considered in this experiment. A total of 15,000 snapshots were taken and evaluated for each test. Snapshots of length 2, 3, 6 and 10 ms were extracted from the recordings. In the acquisition, coherent integration of 1 ms was selected, and 1, 2, 5 and 10 epochs were added incoherently, respectively. The integration time significantly influenced the performance of acquisition and the accuracy of the estimation for the beamforming steering vector. Acquisition was done on each snapshot, followed by the estimation of the beamforming steering vector for each acquired satellite. Lastly, the normalized correlation matrix $C_{\mathrm{norm}}$ was calculated from the array steering vectors (see Section 3).

In the analysis of the data the following detection metrics were evaluated:

- the steering vector angle between a pair of satellites, i.e., the creation of the detection matrix D (see Section 3),
- the fraction of detections in the detection matrix D (see Section 4.1),
- the Eigenvalue maximum-to-mean ratio of the normalized correlation matrix $C_{\mathrm{norm}}$ (see Section 4.2),
- the number of clusters of the Louvain algorithm based on the normalized correlation matrix $C_{\mathrm{norm}}$ (see Section 4.3),
- the community size maximum-to-mean ratio for the Louvain algorithm based on the normalized correlation matrix $C_{\mathrm{norm}}$(see Section 4.3), and
- the modularity *Q* from Louvain clustering based on the normalized correlation matrix $C_{\mathrm{norm}}$(see Section 4.3).

For each detection metric, the probability density function (PDF) p(x) was calculated from the recorded data and compared. This allowed for the statistics of the metrics to be evaluated. To generate the PDF, a histogram from every detection metric was first calculated. The histogram was then normalized according to the bin-widths to obtain the PDF. Since a histogram is limited by a finite resolution, it is a discontinuous approximation of the true PDF. This is shown by the “step” like function in some of the plots. The histogram bin-sizes were changed depending on the metric used. From the PDF the mean μ and standard deviation σ can also be determined [51]:

(37) $$\mu =E\left[X\right]=\int_{-\infty }^{\infty }xp\left(x\right)dx\;,$$

(38) $$\sigma =\sqrt{E\left[X^{2}\right]-E{\left[X\right]}^{2}}=\sqrt{\int_{-\infty }^{\infty }x^{2}p\left(x\right)dx-\mu^{2}}\;,$$

where $E\left[\cdot \right]$ is the expected function for the random variable *X*. Both the mean μ and standard deviation σ are shown in the figures to allow for comparison of the detectors. This supports the analysis by summarizing the probability densities.

Depending on whether the spoofed data or the real data had higher mean values, the left- or right-CDF was calculated for each metric. The CDFs were generated from the associated PDFs. The left-CDF $c_{l}\left(x\right)$ is obtained by integrating the PDF p(x) from the left:

(39) $$c_{l}\left(x\right)=\int_{-\infty }^{x}p\left(x\right)dx\;.$$

On the contrary, the right-CDF $c_{r}\left(x\right)$ is obtained by integrating PDF p(x) from the right:

(40) $$c_{r}\left(x\right)=\int_{x}^{\infty }p\left(x\right)dx\;.$$

Note that the left-CDF is always a monotone increasing function, whereas the right-CDF is always a monotone decreasing function. The left- and right-CDFs were used, as this allowed for a simple comparison of the distributions, and to illustrate how similar or disjointed the distributions were. Further, it allows for the separation between the distributions to be clearly visualized and analyzed. Lastly, a detection threshold was set for each metric to optimize correct detections, under the assumption, that a spoofing attack and no attack are equally likely. Note that this assumption is only valid for the test data and does not represent the likelihood of a spoofing attack in the real world, but is still adequate for research and development purposes. In this case, the optimal threshold λ would be the value by which the left- and right-CDFs for the spoofing and real data distributions are equal:

(41) $$c_{l}^{\mathrm{spoof}}\left(\lambda \right)=c_{r}^{\mathrm{real}}\left(\lambda \right)\;,\;\;\mathrm{or}$$

(42) $$c_{l}^{\mathrm{real}}\left(\lambda \right)=c_{r}^{\mathrm{spoof}}\left(\lambda \right)\;,$$

where ${\left(\cdot \right)}^{\mathrm{real}}$ denotes the real data distribution and ${\left(\cdot \right)}^{\mathrm{spoof}}$ the spoofed data distribution. The assignment of the left- and right-CDF depends on the metric used: for some the spoofed distribution is larger than the real-data distribution, for others it is the opposite. This was indicated by the results. For some metrics, the detection values of others were used; hence, the analysis was done sequentially.

To illustrate some observed effects and limitations in the data, results, where only two of the six antenna elements were used for acquisition and beamforming, are also shown. However, this is not the focus of this article and is not provided within the analysis.

## 6. Results and Analysis

First, the recordings were analyzed with a standard GNSS software-defined radio (SDR) to estimate the power level of the recorded signals. In this analysis, each antenna channel was analyzed separately (i.e., no beamforming is applied). The roof recording received the signals between 39 and 45 dBHz, and the laboratory spoofing setup received the signals between 46 and 48 dBHz. This shows that the spoofing scenario has 3 to 7 dB signal power, which is representative of many spoofing attacks; however, this also directly impacts the detection capability of any method. Secondly, the number of acquired satellites by the snapshot receiver was also analyzed. For the roof recording, 5 to 8 satellites were acquired, whereas 10 to 12 were acquired by the spoofing scenario. This also proved to have an effect on the performance of the detectors.

**Figure 6:**
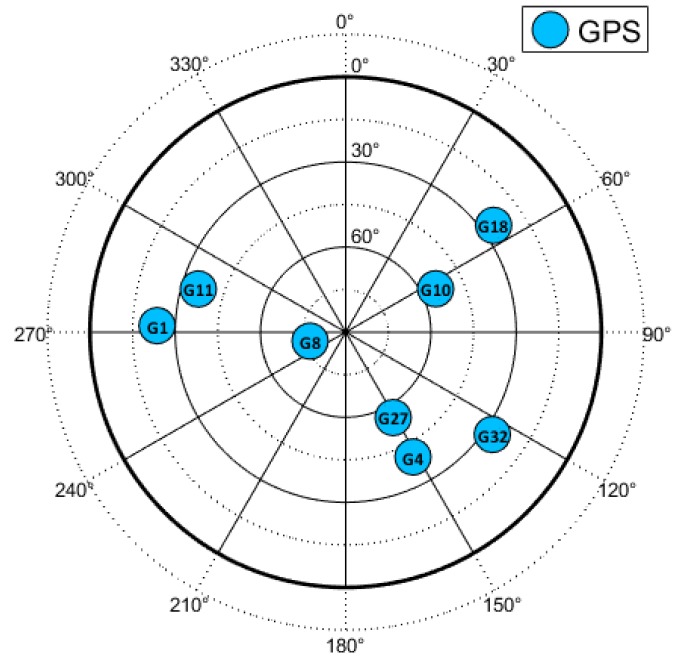
Sky-plot of the open-sky recording. ©IEEE. Reprinted, with permission, from [22].

The sky-plot with visible GPS satellites is shown in Figure 6.

The detectors were evaluated at different values for the steering vector angular threshold $\xi_{\mathrm{Th}}$. This allowed for the performance of the detector to be determined at different thresholds. Open-sky real-world recordings using the array were used.

### 6.1. Single Value Comparisons

**Figure 7:**
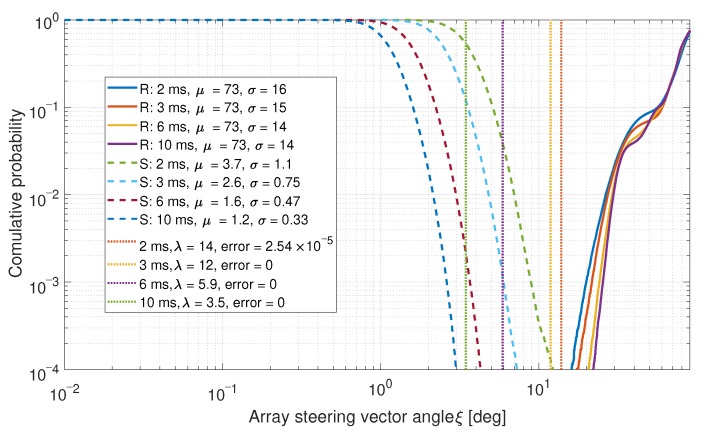
CDF comparison for the steering vector angles between satellites for a six-element array.

The PDF of the values in the upper right triangle of the correlation matrix $C_{\mathrm{norm}}$ was calculated to determine the difference between the real and spoofed measurements. The left CDF is calculated for the real data (denoted by “R” in the legend) and the right CDF is calculated for the spoofed data (denoted by “S”). The CDFs with the mean μ and the standard deviation σ for each distribution, as well as an optimal threshold λ with error probabilities are shown in Figure 7. For a full analysis of this detector method, refer to [22].

**Figure 8:**
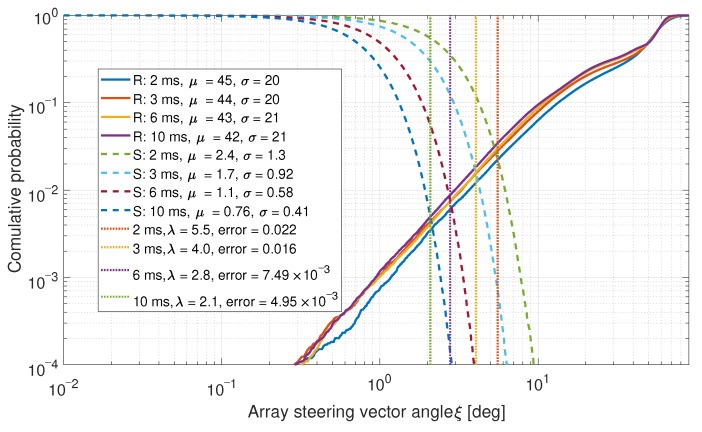
CDF comparison for the steering vector angles between satellites for a two-element array.

As a comparison, the same data for a two-element array (i.e., only using two-elements from the six-element array.) is shown in Figure 8.

The spoofed signals have lower mean μ and standard deviation σ values in both cases. This is expected as the steering vectors for these signals are similar. In both cases, low error probabilities were achieved. Often, no errors were observed at all.

**Figure 9:**
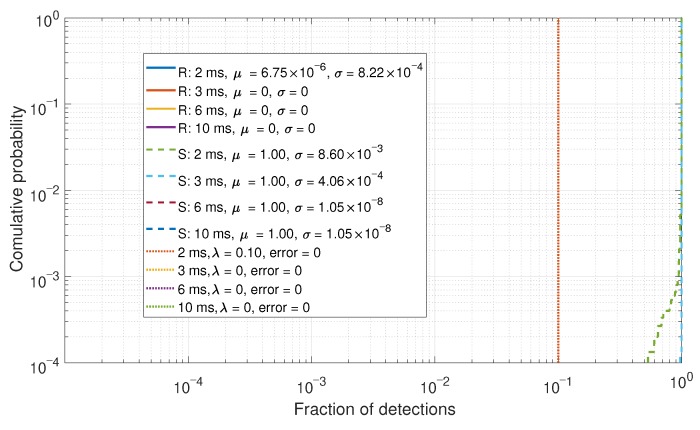
CDF comparison for the percentage of detections for a six-element array.

**Figure 10:**
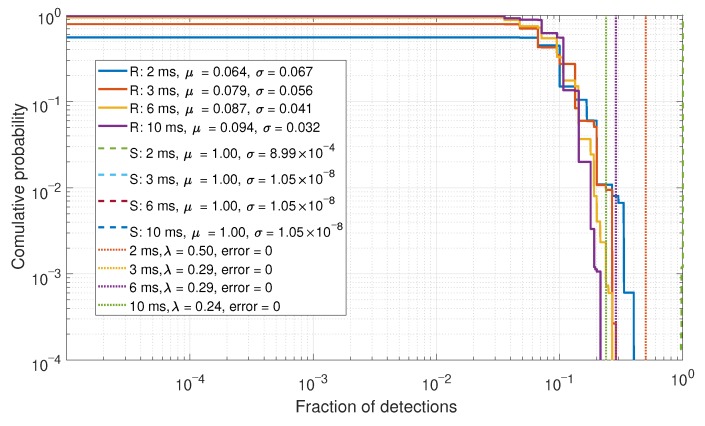
CDF comparison for the percentage of detections for a two-element array.

The fraction of detections based upon the decision threshold of Figure 7 is shown in Figure 9. As there are few to no errors in Figure 7, these results are consequently sparse. However, when the error probabilities are higher, then a spread of data can be observed, as shown in Figure 10.

In this simple case of comparing the fraction of detections, a system of detectors has shown benefits to improve overall performance. However, the performance of the fraction of detections is dependent on the detection performance of the previous stage. Further, such an approach requires the setup of two detector thresholds; hence, increasing the complexity of tuning and calibration for such an approach.

### 6.2. Eigenvalue Comparisons

**Figure 11:**
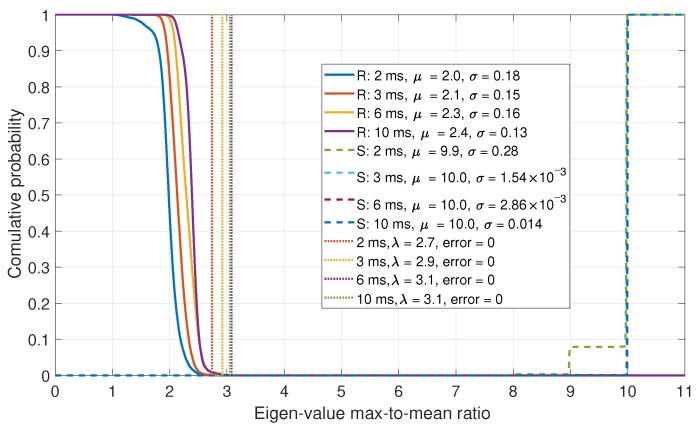
CDF comparison for the maximum-to-mean of the Eigenvalues for a six-element array.

In this section, the Eigenvalues of the correlation matrix $C_{\mathrm{norm}}$ were evaluated. As the number of steering vectors is dependent on the number of acquired satellites, the number of Eigenvalues and vectors are inconsistent. As such, the ratio of the maximum-to-mean Eigenvalues was evaluated. The CDFs for the maximum-to-mean Eigenvalues are shown in Figure 11.

From the Eigenvalue results, it can be shown that a good separation between the real and spoofed data is achieved. The maximum-to-mean ratio exploits the fact that the signal subspace of the Eigenvectors is small for a spoofing scenario, as all of the array steering vectors are similar. This detector also achieves perfect detections, like what was observed by the fraction of detections approach. However, this method only requires a single threshold to be set, making it simpler to tune and to optimize. The disadvantage of the Eigenvalue approach is that it requires more processing than the fraction of detections approach.

### 6.3. Modularity Comparisons

**Figure 12:**
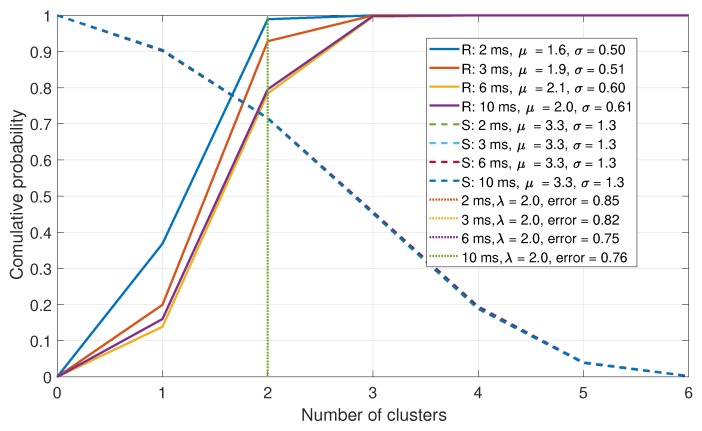
CDF comparison for the maximum-to-mean of the Eigenvalues for a six-element array.

In this section, the metrics that are based upon the Louvain algorithm applied to the normalized correlation matrix $C_{\mathrm{norm}}$ are presented. First, the number of clusters is evaluated. The CDFs of the number of clusters after clustering is shown in Figure 12. In a spoofed case it is assumed that fewer clusters would be generated, as is seen in the figure; however, the difference is insufficient to produce adequate detection results.

**Figure 13:**
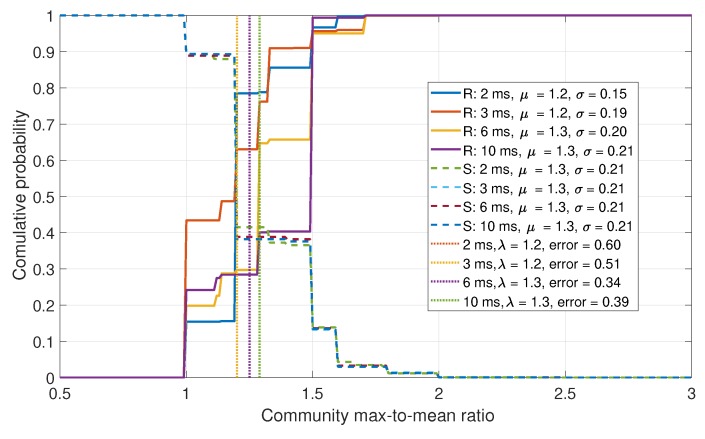
CDF comparison for the maximum-to-mean of the Eigenvalues for a six-element array.

The next approach is to investigate the difference in community sizes. The CDFs for the community are shown in Figure 13. In a spoofed scenario, it is assumed that fewer, larger communities would exist. This was observed in the data, but as with the previous case, insufficient isolation between the real and spoofed data was obtained.

**Figure 14:**
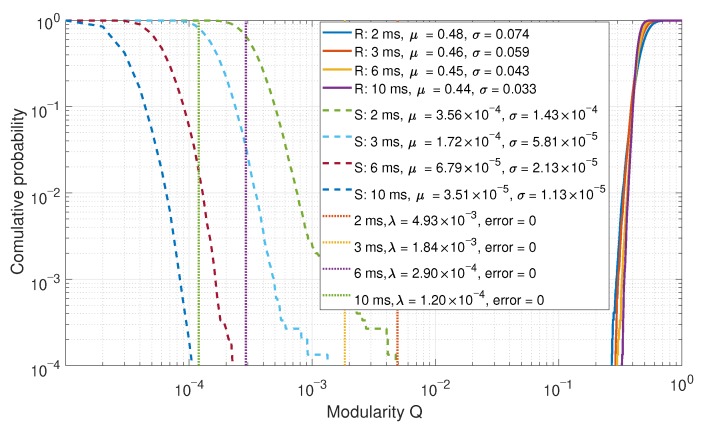
CDF comparison of the modularity after clustering for a six-element array.

Lastly, the modularity of the clustering was observed. The CDFs for the modularity are shown in Figure 14. In a spoofed case, the communities would be clustered more densely, resulting in lower modularity. This is consistent with the results.

The results of using modularity also achieved perfect detection, similar to what was observed with the fraction of detections and the Eigenvalue maximum-to-mean ratio approaches. This approach is an iterative method; hence, it potentially requires more processing than Eigenvalue methods. However, the Louvain algorithm is optimized for efficient processing [48] on large data sets. Therefore, this would not present an issue.

## 7. Discussion of Results

As the integration time used for acquisition increases, the estimation of the array steering vector improves. The variance of the array steering vectors is consequently reduced, resulting in an improved representation of the normalized correlation matrix $C_{\mathrm{norm}}$. In the spoofed case, the correlation matrix tends to be a unit matrix I. Therefore, it is expected and observed in all results, that superior performance is achieved with longer integration times.

Using a detector to estimate the spoofing of a pair of satellites resulted in sufficient spoofing detection. However, it only considers two satellites at a time and is sensitive to the spatial relationship between the two satellites. When a system of satellites is observed, better results can be obtained, as what is shown in the system of detectors.

Three metrics have shown to be useful for detecting a spoofed scenario as all three of these have demonstrated perfect detection capabilities for the given scenario. Firstly, the fraction of detections is built upon the detection of a single pair of satellites. This method combines all detections, hence, it is robust against a single false detection through increased redundancy. This method is straightforward to implement but requires the design of two detectors. Secondly, an Eigenvalue decomposition-based detector was evaluated. This metric exploits the fact that similar satellites would result in a sparse signal sub-space of the Eigenvectors. A maximum-to-mean Eigenvalue ratio was used. This method is more computationally expensive than the fraction of detections but requires fewer calibration and detectors to be developed. Thirdly, a clustering approach based upon the Louvain algorithm was presented. This metric observes the similarity of the array steering vectors as edges between communities. This method has similar advantages and disadvantages to the Eigenvalue based metric. All other metrics presented have been found to have inadequate performance but are nonetheless interesting from a research point of view and for evaluation purposes.

The results are compiled from a high number of observations including the use of different integration lengths. Therefore, a high degree of accuracy and confidence in the methods were achieved. However, only a single spoofed and a single real scenario has been evaluated. This is a significant limitation to the presented results, as the authors believe that the number of acquired satellites, the distribution of the satellites within the sky (i.e., are the satellites close to each other), the recording scenario including degradation effects (e.g., multi-path, shadowing, array coupling effects etc.), the received signal strength of the satellites (i.e., the carrier-to-noise density ratio (*C*/*N*_0_)), the antennas used, and the array geometry will have an impact on the results. As such, further studies including additional observations are required to improve the validity of the results.

In the presented results, the complete constellation is either real or spoofed. However, in many spoofing attacks, a mixed constellation of real and spoofed signals is observed. This is not accounted for in the results but it is believed that Eigenvalue and clustering techniques could benefit in a mixed spoofing attack. For future research, it is proposed that mixed attacks are considered such that the spoofing detection metrics could be improved. Lastly, in a mixed spoofing scenario, a secondary goal could be to identify spoofed satellites and to remove these from the PVT solution. Such spoofing mitigation methods are theoretically possible using the metrics presented in this paper; however, the feasibility thereof still needs to be further investigated.

## 8. Conclusions

In this article, blind detection methods for spoofing signals that exploit the spatial diversity of an antenna array were presented. The detection method was implemented in a snapshot receiver and evaluated using open-sky data, recorded with a six-element array. Additionally, a spoofing attack emulation using a splitter instead of an array was used. First, only a pair-wise spoofing detector was presented. Next, several metrics were developed, implemented, and analyzed which consider the entire system of received satellites. These metrics include multiple detectors, Eigenvalue based methods, and clustering methods.

The results have shown that observing a system-level based detector could have significant benefits to spoofing detectors. Three detection metrics resulted in perfect detection capabilities; however, some concerns to why this is not necessarily achievable for all scenarios and experimental setups are highlighted and discussed. This indicates the need for further research in this area. Nevertheless, it is shown in this article that blind spoofing detection methods could yield adequate spoofing detection capabilities and initial results are promising.

For future research, it is suggested to repeat the tests with different satellite constellations to isolate and quantify some effects and variables which can degrade the system. Further, it would be beneficial to validate the performance inside an anechoic chamber or real live tests, as this would improve the legitimacy of a spoofing attack and also allow a mixed spoofing-real satellite set to be recorded. This would also further challenge the existing detection algorithms. Lastly, the identification of the current metrics paths the way for future statistical methods, such as machine learning.
